# Positive regulations of adipogenesis by Italian ryegrass [*Lolium multiflorum*] in 3T3-L1 cells

**DOI:** 10.1186/1472-6750-14-54

**Published:** 2014-06-11

**Authors:** Soundarrajan Ilavenil, Mariadhas Valan Arasu, Jeong-Chae Lee, Da Hye Kim, Mayakrishnan Vijayakumar, Kyung Dong Lee, Ki Choon Choi

**Affiliations:** 1 Grassland and forage division, National Institute of Animal Science, RDA, Seonghwan-Eup, Cheonan-Si, Chungnam 330-801, Korea; 2 Research Center of Bioactive Materials, Chonbuk National University, Jeonju 561-756, Republic of Korea; 3 The United Graduate School of Agricultural Sciences, Tottori University, Tottori-Shi 680-8553, Japan; 4 Department of Oriental Medicine Materials, Dongsin University, Naju 520-714, Korea; 5 Department of Botany and microbiology, College of Science, King Saud University, Riyadh 11451, Saudi Arabia

## Abstract

**Back ground:**

Intramuscular fat deposition in the meat animal is relatively new strategy for developing the meat quality. Fat deposition is largely depending on the adipocyte proliferation and differentiation. Therefore, we investigated the effect of chloroform extract of *L. multiflorum* [CELM] on cell proliferation, lipid accumulation and adipocyte differentiation in 3T3-L1 cells and body weight of mouse.

**Results:**

We identified 6,9-Octadecatrienoic acid, Hexadecanoic acid, 2-hydroxypropanoic acid, butane-2,3-diol and hexane-1,2,3,4,5,6-hexaol in CELM. *L. multiflorum* extract increased the cell viability, lipid accumulation, cell cycle progression and key transcriptional and secretory factors like PPRAγ2, C/CEBP-α, adiponectin, aP2, GLUT-4, FAS and SREBP-1 mRNA expression as compared with control cells. For *in-vivo*, mice administered with CELM significantly increased body weight throughout the experiment periods. Further, the identified fatty acids like 3, 6, 9-Octadecatrienoic acid and Hexadecanoic acid was docked with target protein [PPRAγ2] using HEX 6.12. The least binding energy considered as high affinity with target protein. The maximum affinity with the target protein was observed in the Hexadecanoic acid followed by 3, 6, 9-Octadecatrienoic acid. The binding efficacy of Hexadecanoic acid and 3, 6, 9-Octadecatrienoic acid to the active site of PPAR-γ2 may be enhanced the adipocyte differentiations.

**Conclusion:**

These findings suggest that CELM stimulates adipogenesis via activating the PPARγ-mediated signaling pathway in adipocyte which could be useful for the development of meat quality in animals.

## Background

Intramuscular fat deposition or marbling in meat animals is an important component of uniqueness which influences the eating quality like meat tenderness, juiciness and tastes. The deposition of intramuscular lipid is associated with genetic background, development and nutrition of animals. The cellular development of adipocyte is much important for deposition of intramuscular fat in meat animals, because intramuscular fat accumulation is strongly related to increases of adipocyte proliferation and differentiation which is main regulatory steps in the marbling [1]. The animals possess very good integrated systems for regulating the energy storage and their expenditure. These systems are mainly involved in utilizing the energy storage during excess food available. When the system meets an inadequate food supply, the mobilization of food from the storage place will be progressed by regulating the cellular levels and whole body organisms through its coordination action of circulating hormones and different neural signal from the central nervous system [2].

Adipogenesis is a process of adipocytes cell proliferation and differentiation. When the cells meet positive energy balance, adipogenesis stimulates the storage of energy through the generation of functional fat cells. These processes were achieved in adipocytes [3]. In adipocytes differentiation, fibroblastic like pre-adipocytes were matured into spherical shape adipocytes which contain huge lipid droplets. Specific transcriptional factors such as peroxysome proliferators activated receptor- γ [PPAR-γ], CCAAT/enhancer binding protein- β [C/CEB-β], enhancer binding protein- δ [C/CEB-δ], enhancer binding protein- α [C/CEB-α], Kriuppel like factor and sterol regulatory element binding protein-1[SREBP-1] are mainly involved in promoting the adipogenesis [4]. These adipogenic transcriptional factors have the ability to regulate gene expression. During the adipocytes differentiation, the C/CEB-β and C/CEB-δ, factors are induced immediately by glucorticoids and Insulin hormones. Then it activates the PPAR-γ and C/CEB-α. The final stage of adipocytes differentiation requires the expression genes which are involved in the adipocytes phenotype and its function maintenance via lipid metabolic enzymes. Numbers of genes were involved in the lipid metabolism. They are fatty acid binding protein-4 [aP2] which is responding to PPAR-γ and C/CEB-α. aP2 is a key factor of intracellular fatty acid transport and lipid metabolism. This aP2 is regulated through the PPAR-γ and C/CEB-α. It is identified as the key marker of terminal steps in the adipocytes differentiations [5,6]. The PPAR-γ has ability in modulating the insulin-signaling pathway through up regulation of many factors involved in the signaling cascade from GLUT-4 which enhances the glucose uptake levels by the cells. The adiponectin is a hormone called as adipo-Q or adipocytes complement related protein which is specifically expressed in the adipose tissue during differentiation [7]. This hormone enhances the sensitivity in muscle and liver. It increases free fatty acid oxidation in several tissues including muscle fiber [2,8].

***Lolium multiflorum*** (Italian Rye-grass IRG - poaceae family) is an herbaceous annual, perennial grass is being cultivated in South Korea for production of silage for animals. In South Korea IRG is a common grass for the production of silage because of it possesses the high nutritive values and fast growing nature under this climate. The nutritional quality of IRG varied with other varieties. Farmers purchases many types of forage products from companies to use as a feed for ruminants, but the cost of using a commercial feed is relatively expensive than IRG-based silage is used. Thereby, we investigate the chemical composition of *L. multiflorum* and their effects on adipogenesis using 3T3-L1 cells because adipogenesis is an important step in the intramuscular fat (marbling) accumulation.

## Methods

### Chemicals

Mouse 3T3-L1 pre-adipocytes were purchased from the American Type Culture Collection [ATCC, USA]. Dulbecco modified Eagle medium/high glucose [DMEM], cell viability assay kit [EZ-CYTox, Daeillab Service Co.Ltd.], mRNA extraction kit and RT-PCR kit were obtained from the Daeil Lab services Co., Ltd and Invitrogen Life technology. Other chemicals and analytical grade solvents were procured from Sigma [USA].

### Plant material and extraction

The *L. multiflorum* (poaceae family) was collected during the month of September, 2011 from the farm in National Institute of Animal Science [NIAS], South Korea. The plant was authenticated by a taxonomist at NIAS. The *L. multiflorum* was harvested at flowering stage without root and ensiled. *L. multiflorum* were collected at 40 days after cultivation, dried at 60°C for 3 days and grounded by using an electric grinder to give a coarse powder. The powder obtained [5 kg] was soaked in 10 L of 70% methanol for 72 h at room temperature by intermittent mixing using orbital shaker. After that the mixture was filtered using Whatman filter paper to separate the supernatant and the sediment. The filtrate was concentrated under reduced pressure at 40°C until extraction solvent was completely removed and stored in a refrigerator at 0–4°C for further use in subsequent experiments. A green soluble crude residue was obtained [about 100 g]. Then this extract was subjected into sequential extraction with chloroform, ether and water for further purification and analysis.

### LC/ESI-MS/MS analysis of plant extract

An API 4000 Q TRAP tandem mass spectrometer [Applied Biosystems, Foster City, CA], equipped with an Agilent 1200 series HPLC system [Agilent Technologies] and an electrospray ionization tandem mass spectrometry [ESI-MS/MS] source in positive ion mode [[M + H]^+^], was used to identify the chloroform extract. The analytical conditions of mass spectrometry were described in detail as follows: range, start [100 amu], stop [1,300 amu], and scan time [4.8 s]; curtain gas, 20 psi [N_2_]; heating gas temperature, 550°C; nebulizing gas, 50 psi; heating gas, 50 psi; ion spray voltage, 5500 V; declustering potential, 100 V; entrance potential, 10 V.

### Cell culture

The 3T3-L1 pre-adipocytes were obtained from ATCC [Manassas, VA]. Cells inductions for differentiation from pre-adipocytes to adipocytes were carried out by Choi et al. [9] with slight modification. Briefly, 3T3-L1 pre-adipocyte were seeded in the 6 well at a density of 3×10^4^ cells/well. The cells were plated and maintained for 2 days in DMEM containing 10% fetal bovine serum. The medium was replenished every 48 h. 3T3-L1 pre-adipocytes were induced the differentiation by the addition of DMEM containing 10% fetal bovine serum, 0.5 mM 3-isobutyl-1-methylxanthine, 1 μM dexamethasone, and 1 μg/ml insulin and antibiotics. After 48 h, medium was replaced with DMEM supplemented with 10% fetal bovine serum, and cells were maintained in this medium for at least 10 days after the induction of differentiation. The plates were incubated at 37°C in 5% CO_2_. The effects of chloroform extract of *L. multiflorum* on adipogenesis enhancement were monitored. 10 mg of extract was dissolved in the chloroform for adipocyte experiment. Every two days, the adipocytes were treated with 10, 50 and 100 μg/ml chloroform extract of *L. multiflorum* until the end of the experiment periods [10 days], without *L. multiflorum* extract considered as control cells.

### Cell viability assay

The water soluble tetrazolium [WST; 2[2-Methoxy-4-nitrophenyl] - 3[4-Nitrophenyl]-5-[2, 4- disulfophenyl]-2-H-tetrazolium monosodium salt was used for analysis of viability of 3T3-L1 pre-adipocytes cells. The cells were seeded in the 96 well at a density of 5×10^3^ cells/well. The cells were exposed to the chloroform extract of *L. multiflorum*. It was incubated at the 37°C in 5% CO_2_ incubator for 24 h and then the culture was treated with WST incubated for 2 h. The living cells absorbed the WST then it was converted into an orange colour product. Then intensity of colour was measured at 450 nm using spectra count ELISA reader [Packard Instrument Co., Downers Grove, IL, USA]. Based on the viability test we have selected the chloroform extract of *L. multiflorum* for adipocytes differentiation investigation.

### Oil red O staining for lipid accumulation

Tested cells were fixed with 10% formalin for an hour and then removed the formalin. After that the cells were washed with 60% Isopropanol, and added 3 ml of Oil Red O working solution to the fix cells. The fixed cells were incubated at room temperature for 10 min. After that removed the Oil Red O solution and then washed 2–4 time with distilled water immediately. The cells were photographed using Olympus CKX41 microscope. For quantification, Oil Red O was extracted from wells of fixed cells with 100% isopropanol, and the extracted material was diluted in 100% isopropanol and read at 490 nm using plate reader.

### Flow cytometer analysis for cell cycle

The tested cells were harvested by the trypsinization method and adjusted to a density of 5×10^5^ cells. The cells were washed with ice cold PBS and re-suspended in 1 ml of ethanol. Treated cells were fixed with 25% ethanol incubated at -20°C for overnight. The fixed cells were centrifuged and re-suspended in 300 μl [50–100 μg/ml in 1.12% sodium citrate] of propidium iodine solution [containing 500 units/mL RNase] for 30 min at room temperature. The sample was stored in the dark at 4°C and analyzed the cell cycle using Flow cytometry caliber [Becton Dickinson, San Jose, CA, USA].

### RT-PCR quantification of PPRAγ2, C/CEBP-α Adiponectin, aP2, GLUT-4, FAS and SREBP-1 mRNA expression

The total RNA was extracted according to the manufactures instruction [RNA lipid tissue mini kit, Qiagen USA]. The extracted RNA was measured using UVS-99 Micro volume UV/Vis Spectrophotometer-ACT Gene. A quantity of 1 μg RNA was reverse transcribed using oligo [dT] and III reverse transcriptase is a version of M-MLV RT[superscript III first stand synthesis system for RT-PCR - Invitrogen Life technology]. Real-time PCR was carried out with an ABI 7500 Real-Time PCR System. Target cDNA levels were determined by SYBR green-based real-time PCR in 20 μL reactions containing 10 μL Power SYBR Green Master Mix [Applied Biosystems, Foster City, CA], 10 pmole forward [FP] and reverse primers [RP] [PPAR-γ2 - FP: gtgctccagaagatgacagac, RP: ggtgggactttcctgctaa, C/EBP-α- FP: gcaggaggaagatacaggaag, RP: acagactcaaatcccaaca, Adiponectin- FP: ccgttctcttcacctacgac, RP: tccccatccccatacac, aP2- FP: tgtgtgatgcctttgtgg, RP: tgtgtgatgccttgtgg, FAS-FP: cccagcccataagagttaca, RP: atcgggaagtcagcacaa, GLUT-4- FP: cccacagaaggtgattgaac, RP: ggtggagatgatgacccttt, SREBP-1-FP: gaagtggtggagagacgcttac, RP: tatcctcaaagggctggactg]. Expression was normalized against beta-actin transcript signal.

### Animals

IRC mice [Each 20 g body weight] were obtained from Orient Co., Seoul Korea. They were housed under standardized conditions [at a constant room temperature with alternating 12-h periods of light and darkness and they fed with standard laboratory diet [AIN 93G, Feed lab Co., Korea]. The use of animals in this study was approved by the Laboratory Animal Center (Permit Number: CBU 2012–0039) of Chonbuk National University (Jeonju, South Korea) and all of the experiment was carried out according to the guidelines of the Animal Care and Use Committee of the University.

### Experimental design

Each group consist of 6 mouse for four groups

I. Group I Control mice that received normal food and water for seven weeks

II. Group II Mice received normal food along with chloroform extract of *L. multiflorum* 100 mg/kg of body weight for seven weeks

III. Group III Mice received normal food along with chloroform extract of *L. multiflorum* 200 mg/kg of body weight for seven weeks

IV. Group IV Mice received normal food along with chloroform extract of *L. multiflorum* 300 mg/kg of body weight for seven weeks

After experimental periods, the mice weight was measured.

### Statistical analysis

Numerical data obtained from experiments were expressed as mean [mean ± SEM] standard error. Statistical difference between the Control and experimental cells were analyzed by SPSS/16 software hypothesis testing methods that including analysis of variance [ANOVA] followed by least significance difference test. P values of less than 0.05 were considered to show statistical significance.

### Molecular Docking

PPAR-γ2 PDB format obtained from the RCSB Protein Data Bank [http://www.rcsb.org/pdb]. After that we have identified the possible binding site of - PPRA-γ2 using Q-Site Finder for predicting the ligand binding sites [http://www.modelling.leeds.ac.uk/qsitefinder/]. The conical smile and PDB format of the ligand had been obtained from http://pubchem.ncbi.nlm.nih.gov/, http://cactus.nci.nih.gov/services/translate/. Docking analysis was carried out using HEX 6.12version. The following parameters are considered for docking purpose Correlation type, FFD mode, Grid dimension, and receptor, ligand and twist range. The interaction of ligand with receptor [LIGPLOT] after docking has been obtained from the PDB SUM [http://www.ebi.ac.uk/pdbsum/].

## Results

### Effect of *L. multiflorum* extract on adipocytes viability

**Figure 1:**
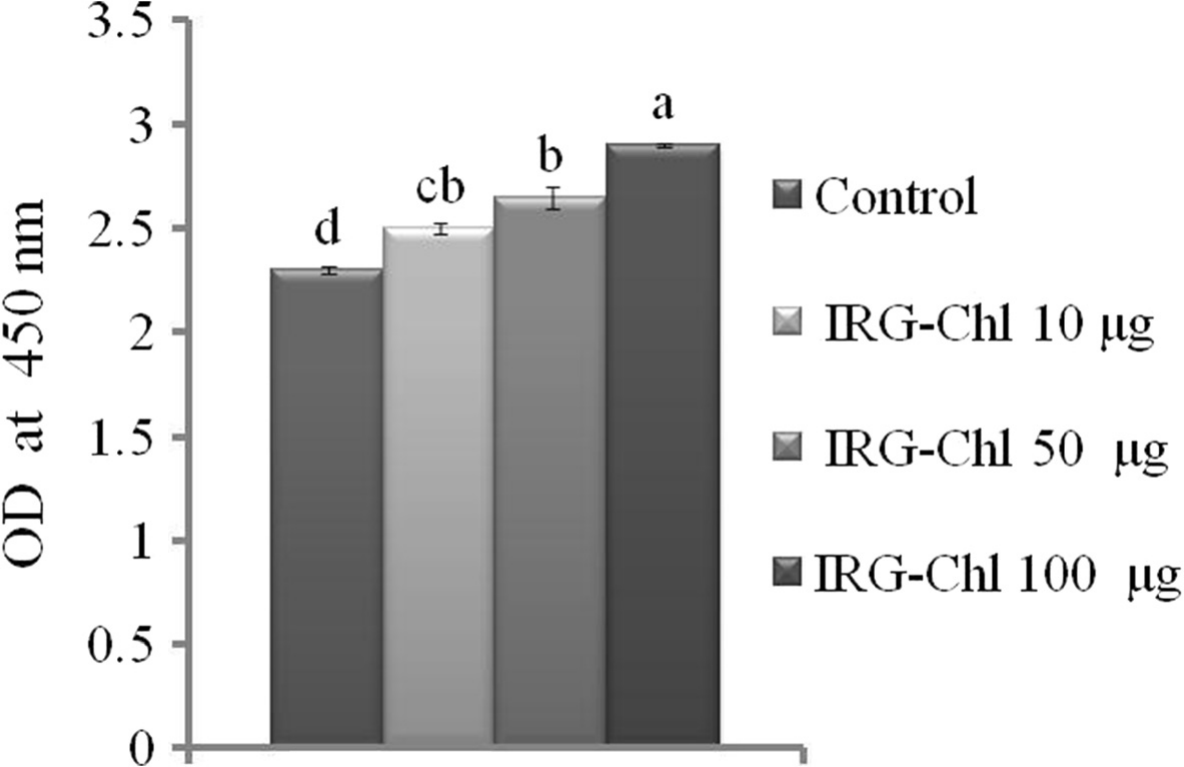
**Effect of *L. multiflorum *extracts on adipocyte viability.** The cells were treated with different concentration of CELM (10, 50,100 μg/ml) for 48 h in 5%CO2 at 37°C. The results represent the mean ± SEM of duplicate experiments. ^abcd^different letters within treatment represent significant difference (p < 0.05).

The chloroform extract of *L. multiflorum*[CELM] shows significantly increases the adipocyte proliferation in a concentration dependent manner. The CELM at a concentration of 100 μg/ml showed maximum adipocyte proliferation as compared with control cells. (Figure 1).

### Analyses of chloroform extract of *L. multiflorum* [CELM] using LC/ESI-MS/MS

**Figure 2:**
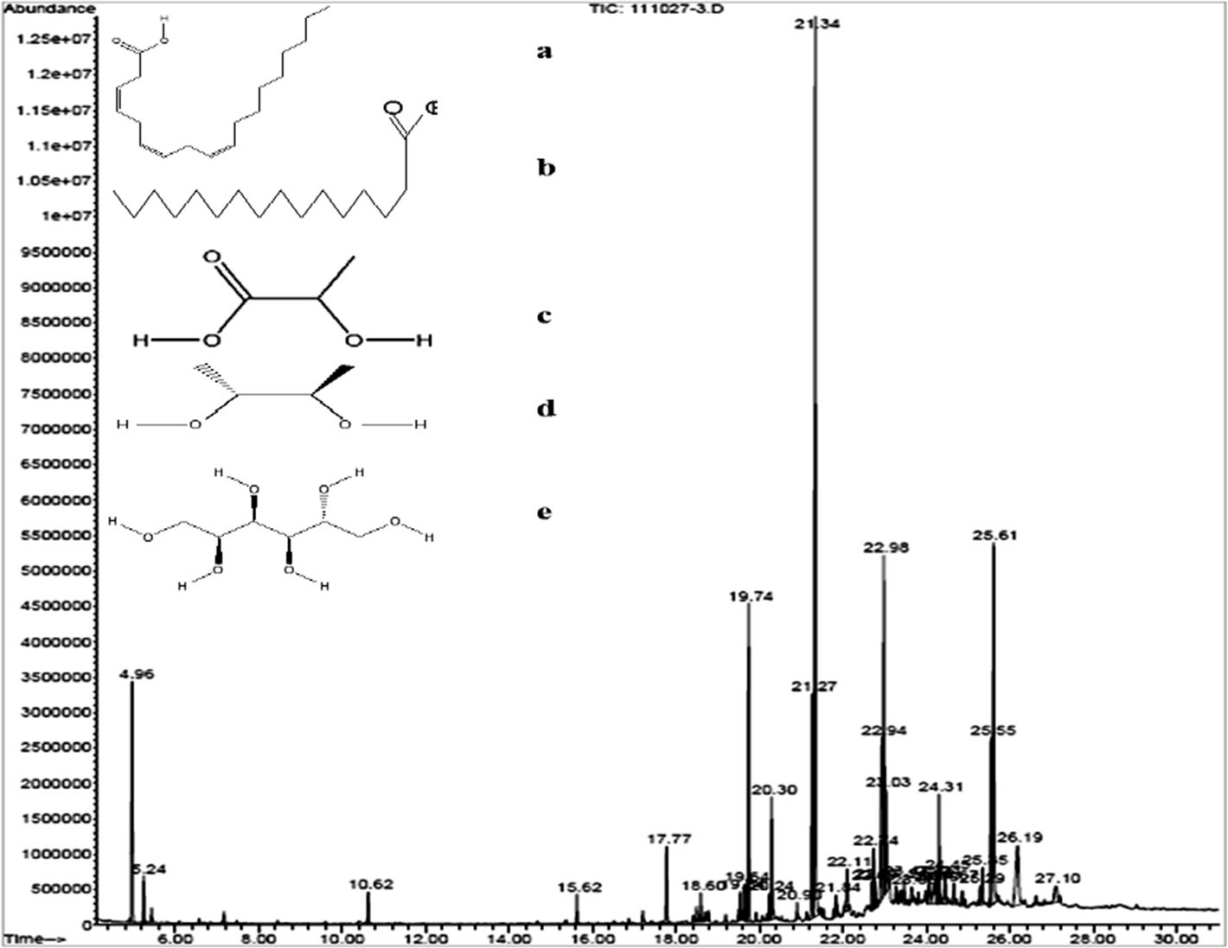
**LC/ESI-MS/MS chromatogram of chloroform extract obtained from *L. multiflorum. *a**; 3,6,9-Octadecatrienoic acid, **b**; Hexadecanoic acid, **c**; 2-hydroxypropanoic acid, **d**; butane-2,3-diol, **e**; hexane-1,2,3,4,5,6-hexaol.

The MS spectral data led to the identification of 3,6,9-Octadecatrienoic acid, Hexadecanoic acid, 2-hydroxypropanoic acid, butane-2,3-diol and hexane-1,2,3,4,5,6-hexaol as the major compounds in the chloroform extract (Figure 2).

### Effect of chloroform extract of *L. multiflorum* [CELM] on adipocyte differentiation

**Figure 3:**
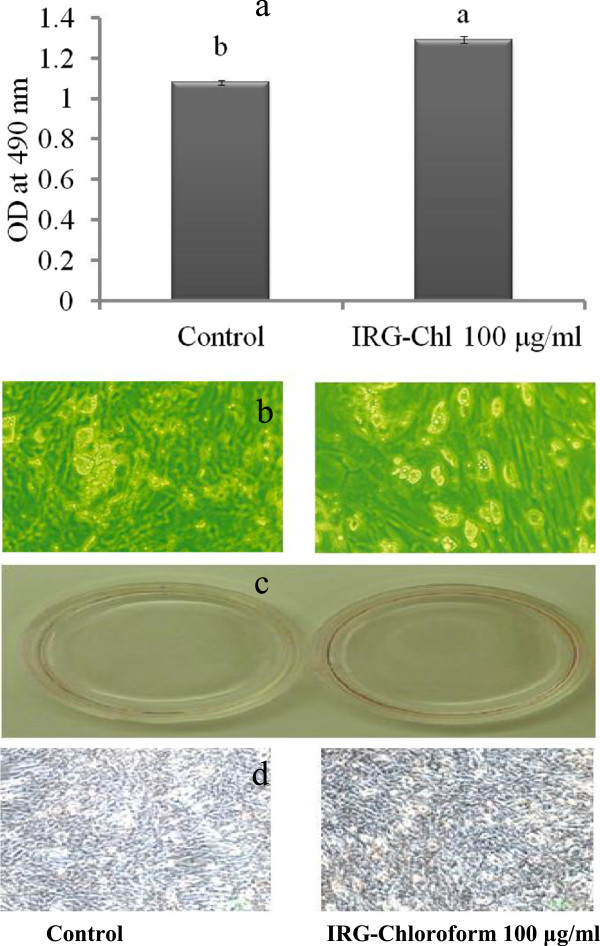
**Effect of chloroform extract of *L. multiflorum *(CELM) on adipocytes differentiation.** 3; Quantification of lipids in adipocytes, 3; Microscopic view of differentiated adipocyte, 3; Oil red O staining of lipid accumulations in cytoplasm of the adipocyte. The results represent the mean ± SEM of duplicate experiments. ^abcd^different letters within treatment represent significant difference (p < 0.05).

**Figure 4:**
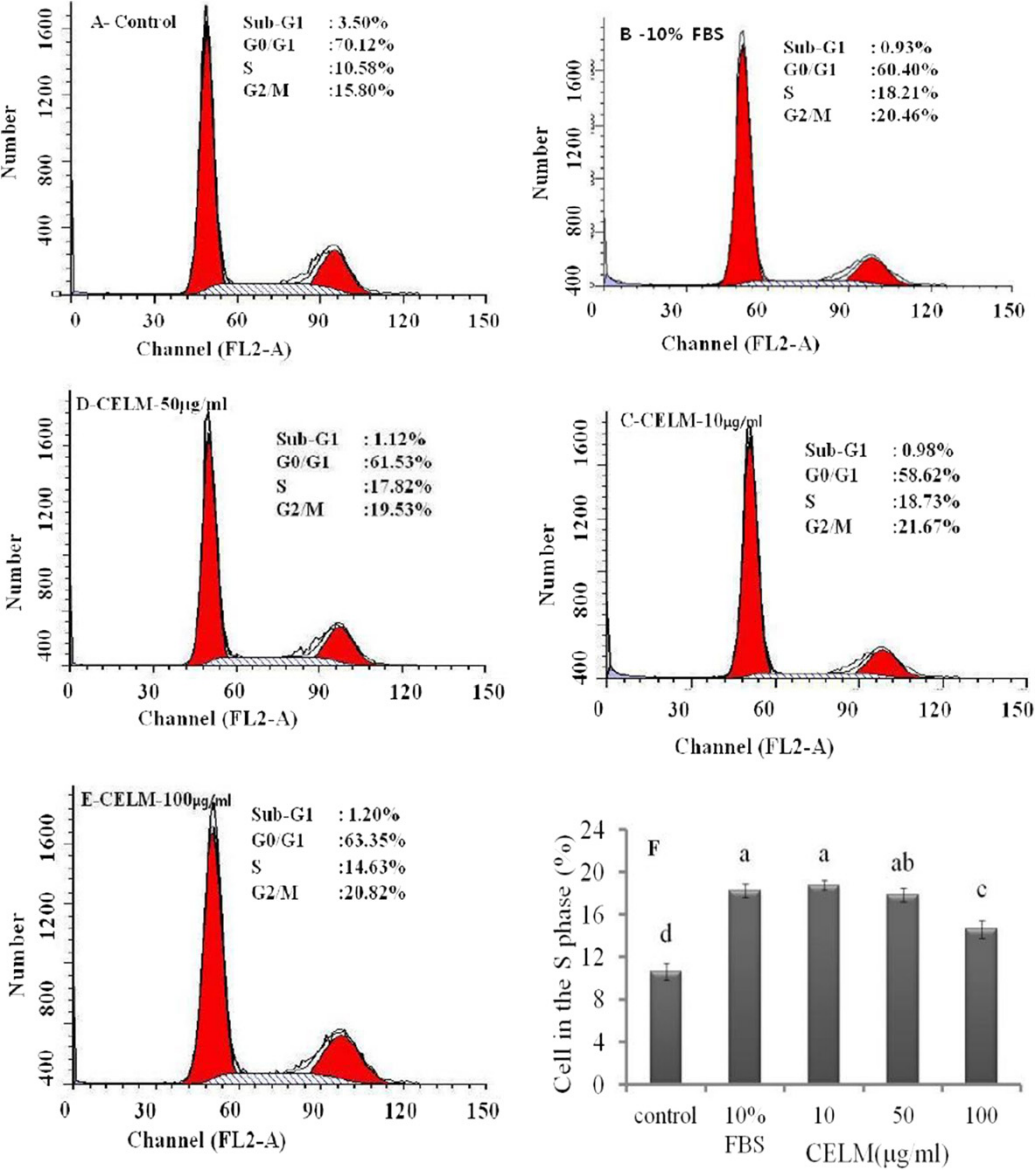
**The adipocyte were treated with different concentration of CELM (C-10, D-50,E-100 μg/ml) for 24 h; B- the cells incubated same times in the 10% FBS as positive control; A- Control cells.** The cell cycle progress was investigated using a flow cytometer; **F**- cell population in the S phase of experimental cells was calculated from triplicate experiments. ^abcd^different letters within treatment represent significant difference (p < 0.05).

After two days of confluence, 3T3-L1 pre-adipocytes were treated with CELM at concentrations of 10, 50 and 100 μg/ml. Results indicated that the pre-adipocytes were differentiated into the adipocyte in a concentration dependent manner. The maximum differentiation was observed at a concentration of 100 μg/ml (Figure 3d). The morphological changes were caused by the accumulation of intracellular lipids in the cytoplasm of the adipocyte. The intracellular lipid content in adipocyte was significantly increased by the cells treated with CELM as compared with control cells. Oil red O staining as an evidence for accumulation of lipids in the cytoplasm of adipocyte (Figure 3a-b and c). Flow cytometry analysis report revealed that the CELM promotes the cell cycle progress. The histogram results revealed that the increased the cell population in the S phase at all the concentration of CELM (Figure 4 a-f).

### Effect of CELM on PPRAγ2, C/CEBP-α Adiponectin, aP2, FAS, GLUT-4 and SREBP-1

**Figure 5:**
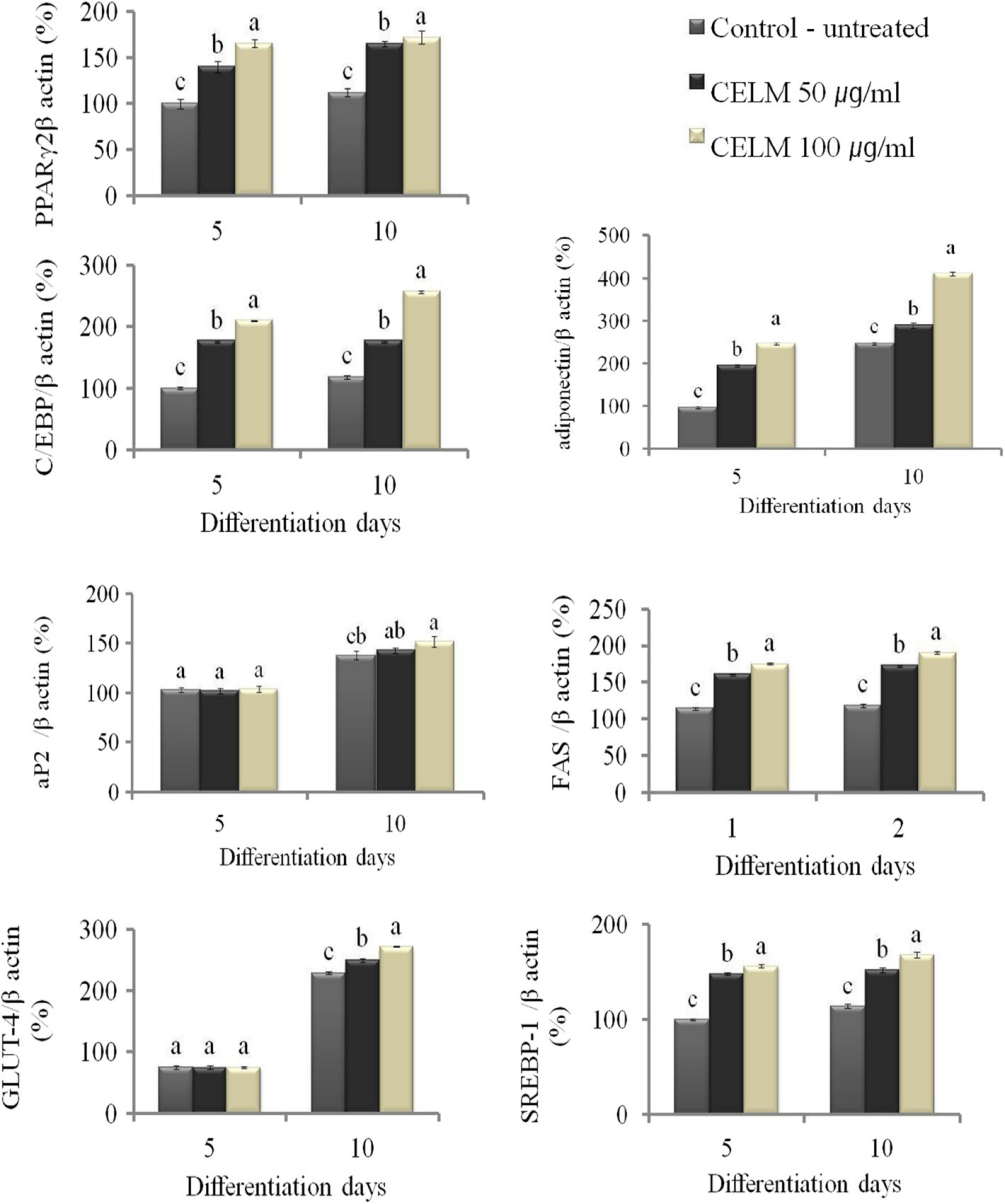
**shows quantification target expression of PPAR- γ2, C/EBP-α, adiponectin, aP2, FAS GLUT-4and SREBP-1 transcript level in experimental adipocyte.** The results represent the mean ± SEM of duplicate experiments. ^abcd^different letters within treatment represent significant difference (p < 0.05).

The adipogenic and lipogenic genes such as PPARγ2, C/EBP –α Adiponectin, aP2, Fatty acid synthase [FAS], GLUT-4 and SREBP-1 was analyzed by qPCR. The expression of PPARγ2, C/EBP-α, Adiponectin, FAS and SREBP-1 mRNA was increased in concentration of 50 and 100 μg/ml of CELM on the 5^th^ and 10^th^ day of differentiation as compared with control cells. For aP2, no changes were observed on the 5^th^ day of differentiation at all the concentrations. But, on the 10^th^ day shows significant increases of aP2 mRNA transcripts as compared with control cells. There is a no significant change of GLUT-4 mRNA expressions was observed in the CELM treated cells at concentrations of 50 and 100 μg/ml on the 5^th^ day as compared with control cells. However, significant increases of GLUT-4 mRNA expressions were observed in the CELM treated cells at concentrations of 50 and100 μg/ml on the 10^th^ day of differentiation (Figure 5).

### Effect of CELM on body weight of mouse

**Figure 6:**
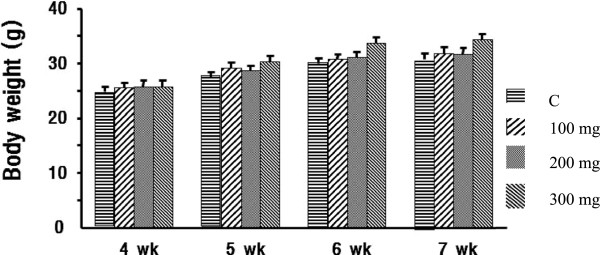
**Effect of extract on the body weight of mice in control and experimental groups.** C: control, 100 mg chloroform extract, 200 mg chloroform extract, 300 mg chloroform extract.

The mice administered with three different concentrations [100, 200 and 300 mg/kg of body weight] of CELM for seven weeks after that the mice weight were measured. Results indicated that the CELM enhanced the body weight of mice from beginning to end of experiment periods (Figure 6). It shows the CELM may be stimulating the adipogenesis and increased the body weight of mice. This result concurrence with the above said molecular investigation.

### Molecular docking

**Figure 7:**
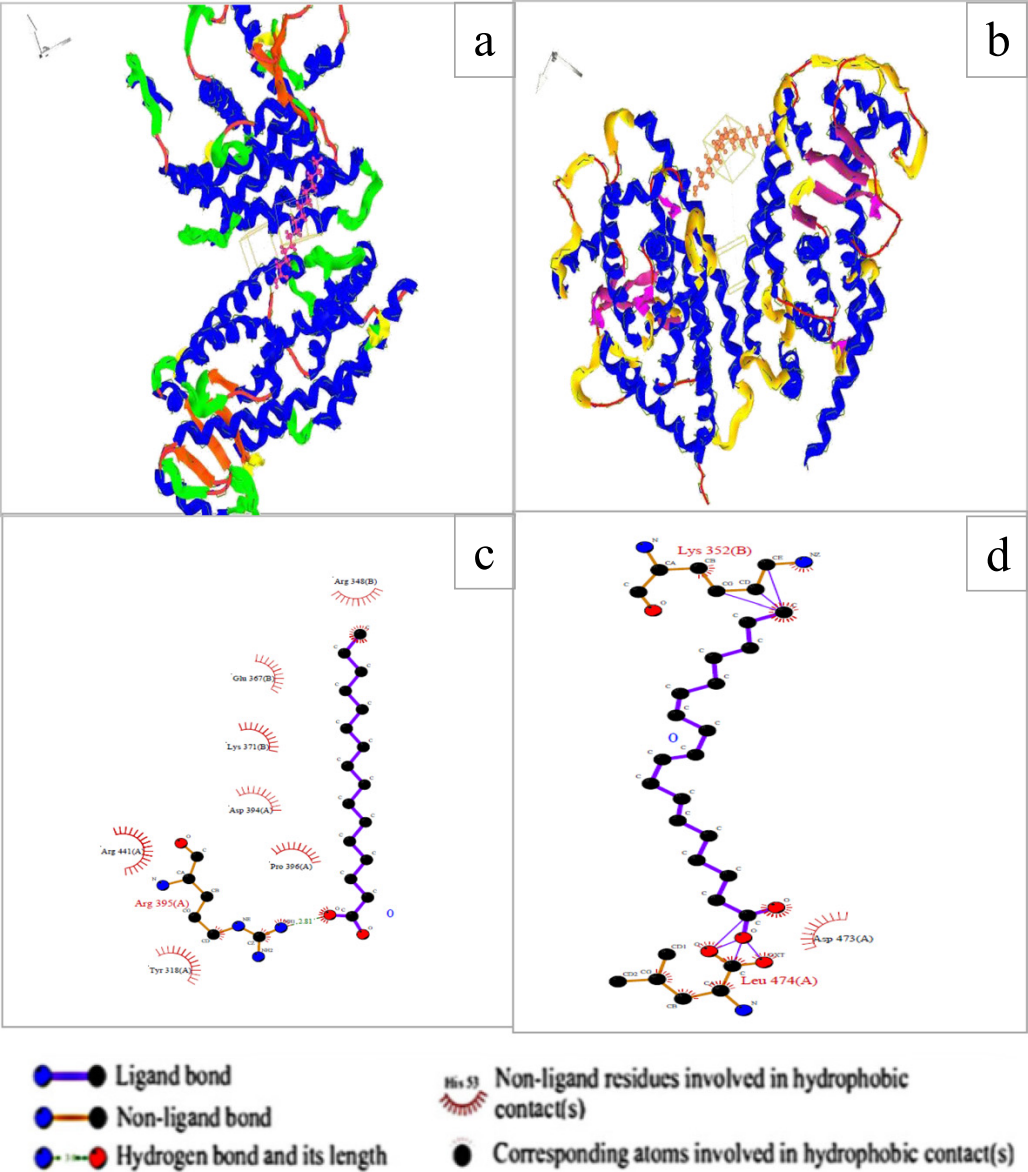
**Docked orientation of fatty acids with target active site protein (PDB ID-3adx) and their run ligplot. a** - Docked orientation of Hexadecanoic acid with active site of PPARγ2, **b** - Docked orientation of 3, 6, 9-Octadecatrienoic acid with active site of PPARγ2. **c** – run ligplot of Hexadecanoic acid with active site of PPARγ2, **d** - run ligplot of 3, 6, 9-Octadecatrienoic acid with active site of PPARγ2. It shows ligand interaction with PPARγ2 by hydrogen bond and non ligand residues involved hydrophobic contacts.

LC-MS analysis revealed that five major compounds such as 3,6,9-Octadecatrienoic acid, Hexadecanoic acid, 2-hydroxypropanoic acid, butane-2,3-diol and hexane-1,2,3,4,5,6-hexaol present in the CELM. Among these, two are fatty acids. Generally, many fatty acids enhance the adipogenesis and lipogenesis. In that aspect, we docked these two fatty acids with adipogenic main key transcriptional factor PPAR-γ2 using HEX 6.12 docking software. The target protein structure was docked with 3, 6, 9-Octadecatrienoic acid, Hexadecanoic acid. The binding energy for 3, 6, 9-Octadecatrienoic acid [-277.64, Emax = -203.42] and Hexadecanoic acid [-273.33, Emax = -176.01]. In docking lowest minimum energy values considered highest affinity with the target protein. We found that the lowest minimum energy in the hexadecanoic acid followed by 3, 6, 9-Octadecatrienoic acid. Hexadecanoic acid strongly binds with Aspartic acid [D-394], Arginine [R-395] and proline [P-396] and Arginine [R-441] of the PPAR-γ2 by hydrogen bond and non ligand residues involved hydrophobic contacts. 3, 6, 9-Octadecatrienoic acid binds with aspartic acid [D-473] and Leucine [L-474] of the PPAR-γ2 by non ligand residues involved hydrophobic contacts. This binding efficacy provides strong evident for CELM enhances the adipogenesis in *in-vitro* cell lines. The enhancement adipocytes differentiations may be due to fatty acids present in the CELM (Figure 7).

## Discussion

Adipose tissue is a very much important for the whole body energy production. It integrates the both central and peripheral metabolic pathways and it regulates the energy balance [10]. Imbalance between energy intake and energy expanse leads to affect the development of the adipose tissue by combination of proliferation and differentiation. During the differentiation periods, the excess of the triglycerides was accumulated in the existing adipose tissue. Our results showed that CELM treatments was increased the proliferation of 3T3-L1 cell as compared with control cells. Han et al., 2009 [11] reported that the protocatechuic acid from aplpinia oxyphylla stimulates the proliferation of adipose tissue derived stromal cells. For differentiation of adipocyte, we observed the CELM promotes the intracellular accumulation of lipids in adipocyte as compared with control cells. It is indicated that the CELM considered as a potent stimulator for adipogenesis. The flow cytometer result revealed that the CELM increased the cell populations in the S phase as compared with control cells. The adipocyte specific transcriptional factor C/EBP-α and PPRAγ2 was increased in adipocyte by the CELM treatment as compared with control cells at concentrations of 50 and 100 μg on the 5th and 10th day. C/EBP-α and PPRAγ2 plays an important role in the adipocyte differentiation because of its key transcriptional factors for other adipogenic genes [12]. C/EBP-α is also involved for stimulating and maintaining the PPRAγ2 expression during the adipogenesis [13]. C/EBP-δ and C/EBP-β are promoters for the key transcriptional factors PPRAγ2 and C/EBP-α. Our results claimed that the increase of PPRAγ2 expression may continuously activate the adipose tissue differentiation by directly acting on the C/EBP-α which creates a positive feedback loop. In addition, C/EBP-α stimulate the genes that are involved in insulin sensitivity, lipogenesis, lipolysis and other encoding genes such as GLUT-4, ap2, lipoprotein lipase [LPL], 1 acyl glycerol-3- phosphate acyltransferase[AGPAT2], perilipin, fatty acid synthase [FAS] and some secreted factors like adiponectin and leptin [14,15]. The CELM may be joined with transcriptional factor to promote the differentiation of the adipocyte. It is consistent with previous reports; the mulberry leaf extract strongly involved the adipogenesis enhancement by stimulating the differentiation of 3T3-L1 pre-adipocyte into adipocyte by enhancing the specific transcriptional factor such as PPRAγ2 and C/EBP-α [16].

Adiponectin is a secretory factor in the adipocyte which is exclusively synthesized and regulated during the adipocyte differentiation [17]. It acts as an autocrine factor in adipose tissue by triggering the gene expression responsible for adipogenesis [18]. In the present investigation, the CELM increased the lipid accumulation in the adipocyte. It’s confirmed by the Oil Red staining method. It may due to increases of adiponectin in the adipocyte by the CELM treatment during the differentiation. It is also predicted that hormones may be directly involved to enhance the intracellular lipid accumulation in the adipocyte. Similarly, the mulberry leaf extract enhances the adiponectin secretion in the differentiated adipocytes by stimulating insulin sensitivity and lipid accumulation. Therefore, our results confirmed that adiponectin could contribute directly to adipose tissue remodeling by increasing many numbers of the adipocytes [16].

Fatty acid binding protein aP2 is present in adipose tissue which is an important protein involved in the regulation of intracellular metabolism and transport of fatty acids. During the adipocyte differentiation, the expression of aP2 level was observed in adipose tissue and adipogenic cell lines [19,20]. We found that the increased levels of aP2 mRNA expression in CELM treated adipocyte as compared with control adipocyte. This expression may be due to the involvement of CELM on lipid metabolism by binding of the long chain fatty acids. During the differentiation periods the lipogenesis was increased. Oil Red O evidenced that the increased the lipid droplets in the CELM treated cells. Therefore, CELM enhances the lipogenesis via ap2 during the differentiation. The adipocytes synthesize adipocyte specific products such as aP2 an adipocyte specific fatty acid binding protein that has been very much important intermediate marker for the adipocytes differentiation [21]. Fatty acid synthase is the main key enzyme in the lipogenesis pathway [22]. It catalyses all the enzymatic steps involved in the conversion of acetyl CoA, molanyl CoA finally to palmitate. Therefore, FAS enzyme and their mRNA expressions are considered as a key marker for the lipogenesis. Increased level of FAS in the CELM treated adipocytes indicated that the extract enhanced the fatty acid metabolism via FAS. It’s consistent with Oil Red O result that CELM increased the lipid accumulation in the adipocytes.

GLUT-4 plays a major role in the energetic and metabolic activities of the adipocyte by allowing the glucose transportation into the cells by the insulin signal. Activation of the insulin receptor stimulates the large increases of GLUT-4 vesicle exocytosis and endocytosis [23]. PPARγ2 also involved in stimulating the adipogenesis and may regulate the lipogenesis by imparting insulin sensitivity to fat cells in responsive adipose depots [24,25]. CELM may stimulate the insulin sensitivity via PPARγ2. This factor has ability to modulate the insulin signaling pathway through the up regulation of several factors for the signaling cascade for GLUT-4 [26].

SREBP-1 highly expressed in the liver and both white and brown adipose tissues. This ADD-1/SREBP-1 mRNA was significantly higher in the pre-adipocytes compared with other fibroblast. Further, it was increased during the differentiation periods [27]. In addition, PPARγ2 and ADD-1/SREBP-1induction stimulates the expression of several important lipogenenic genes in the adipocyte like fatty acid synthase [28] aceyl carboxylase [29,30], glycerol-3- phosphate acyltransferase [31] and the lipoprotein lipase [29]. This ADD-1/SREBP-1 target genes regulate many important steps in fatty acid metabolism by involving in production of natural fatty acids derived PPARγ2 ligands and activators. ADD-1/SREBP regulates the adipocytes differentiation by controlling the PPARγ2 transcriptional factor [32]. In our results at 50 and100 μg/ml concentration of CELM increased the expression of SREBP-1. Therefore, these results confirmed that the CELM enhances the adipocytes differentiation by SREBP-1 gene expression with PPARγ2 induction. The main role of the SREBP-1 is activating the specific transcription of certain genes responsible for adipocytes differentiation. It also has the capacity to trans activate the promoters of the fatty acid synthase and S14 genes. The human analogue of SREBP-1 involved in the regulation of many genes which are responsible of fatty acid metabolism [33]. ADD-1/SREBP-1c may play important role in the action of insulin to regulate the adipocytes gene expression by stimulating genes that involved in the lipogenesis and reducing the genes which are involved in the fatty acid oxidation [34-36]. For confirmation of *in vitro* results, we carried out the animal experiments, mice administered with CELM through orally for seven weeks. The results clearly confirmed the increases of mice body weight as compared with control mice.

The intake of high fat content is associated with fat mass development through key transcriptional factor PPARγ2 which stimulates the adipogenesis. Addition of saturated, mono saturated, and polyunsaturated fatty acids enhance the adipocytes differentiation. Fatty acids stimulate the porcine adipocytes differentiation and their specific transcriptional and co factor genes [37]. Docosahexaenoic acid increased the PPARγ2 leading to increase expression aP2 and adiponectin. Triglycerides mixture, caprylic acid, very low, low and high density lipoprotein stimulate the proliferation and differentiations of bovine adipocytes [38]. The present investigation, we identified the five major compounds 3,6,9-Octadecatrienoic acid, Hexadecanoic acid, 2-hydroxypropanoic acid, butane-2,3-diol and hexane-1,2,3,4,5,6-hexaol, among these, two are fatty acids. These fatty acids were docked with main adipogenic transcriptional factor PPAR-γ2 using HEX docking software 12 because of many reports claimed that the fatty acids enhance the adipogenesis. This docking result also strongly supports both *In-vitro* investigations. The highest affinity with target protein active site is hexadecanoic acid followed by 3, 6, 9-Octadecatrienoic acid.

## Conclusions

Intramuscular accumulation of fat or marbling in meat animals are much important to contribute significantly for developing the quality of meat. The accumulation of intramuscular fat is largely depend on adipocyte proliferation and differentiation because of adipocyte fill the lipids in the cells through a differentiation process and it’s an important regulatory process in the deposition of marbling. Present study, CELM strongly enhances the adipocyte proliferation, lipid accumulation and differentiation through activation of PPAR-γ2 and C/EBP; subsequently it activates the, Adiponectin, aP2, FAS, GLUT-4 and ADD-1/SREBP-1 and Fatty acid synthase. Therefore we suggest that the CELM induces the lipid accumulation through stimulating PPARγ2 that could be useful to make a plan to enhance the meat quality in animals.

## Competing interests

There are no conflicts of interests between the authors.

## Authors’ contributions

SI MVA DHK MV performed the experiments. HSP KDL helped in interpretation of the results and helped draft the manuscript. JCL KCC supervised and analyzed to draft the manuscript. All authors read and approved the final manuscript.
